# Expediency vs. Principle

**Published:** 1896-07

**Authors:** 


### EDITORIAL DEPARTMENT.

F. E. DANIEL, M. D., Editor.
S. E. HUDSON, M. D., Managing Editor.
A. J. SMITH. M. D., Galveston, Associate Editor.

EDITORIAL STAFF:

PROF. J. E. THOMPSON, M. D., Texas Medical College, Galveston; Surgery.
PROF. WM. KEILLER, M. D., Texas Medical College, Galveston; Obstetrics and Gynecology.
PROF. DAVID CERNA, M. D., Texas Medical College, Galveston; Therapeutics.
PROF. A. J. SMITH, M. D., Texas Medical College, Galveston; Medicine
DR. R. H. L. BIBB, City of Mexico; Foreign Correspondent.

Published Monthly at Austin, Texas, by Drs. Daniel and Hudson. Subscription price $2.00 a year in advance.

Eastern Agents: The Monihan-Fairchild Special Agency, 24 Park Place, New York City.

Official organ of the West Texas Medical Association, the Houston District Medical Association, the Austin District Medical Society, the Galveston County Medical Society, and several others.

### EXPEDIENCY VS. PRINCIPLE.

In the matter of seeking medical legislation, the Texas State Medical Association has not only given up the fight against homeopaths, so long and so vigorously waged at the hands of able committees, but, as will be seen by its action at Fort Worth in adopting a bill which provides almost equal representation for them on a board of medical examiners, it has unconditionally surrendered to them. The Association now proposes to take them into loving embrace as friends and allies; and thus reinforced, to continue the war against all other quacks.

Year after year every effort at medical legislation has been defeated, as we have before stated, and as is well known, by these people. They have cried "persecution," and managed to get the sympathy of the law makers. The war was long and bitter, but the white flag has been run up by the "regulars."

For our part—regarding them as I do, as outside of the profession, as not being physicians in any sense of the word—I do not concede to homeopaths the right to be considered at all in the framing of such a law as is needed and heretofore sought; and this position should have the support of all who recognize the code of ethics as their guide. The code does not recognize any "school of medicine," and especially forbids consultation with those "adhering to any dogma, and calling themselves anything but physicians."

The Association now comes out and not only openly recognizes these people as a separate "school of medicine," thereby tacitly consenting to the stigma fixed upon us by Hahneman, as "a school" of "allopaths;" recognizing them as physicians, having equal rights with physicians; as legitimate competitors for practice; not equal, but superior in sagacity and influence to the medical profession of Texas (as far as represented by the State Association), but acknowledges that they are "too much" for them, notwithstanding the relative strength, numerically, of the homeopaths; and admits that there is no use in fighting longer for medical legislation, so long as they, the homeopaths, are opposed to it. And the Association now proposes, as stated, to join forces with them, and attempt to secure with their aid what they have not been able to get with their opposition,—a bill to "*suppress quackery*."

And the *Texas Medical News*, edited by two of the signers of the address alluded to in our June number (two of the signers being at the time on the State Association's Committee on Legislation), now backs the proposition, and makes a special plea for the homeopaths—on the score of "justice" and "expediency."

There is one thing in being defeated; one can, without humiliation, lay down arms, and, even with dignity and self-respect, surrender to superior skill, tactics or numbers, but it is quite another thing to truckle to the enemy and lick the hand that smote you.

The State Association, and, in this instance, its mouthpiece, the *Texas Medical News*, have not only given up the fight, but have surrendered the principle upon which it was made. They have done more:—by the action of the former, and the humiliating confessions made in the May number of the *News*, they have given courage and moral support to the homeopaths which will render them still more blatant and presumptuous; and having obtained now the recognition they have so long fought for, their next demand will be equality and full fellowship in consultation room and in Association meetings.

It is very desirable to have a law that will prevent the ignorant and unqualified, of whatever class, from attempting to practice medicine as well as the shrewd imposter, and according to the writer's way of thinking, there are more ignorant pretenders and well posted frauds sailing under the guise of "homeopathic physicians,"—a misnomer,—a moral impossibility,—a contradiction in terms, in fact,—than all others combined; but if we can not have it without the aid of those whom it would, in a large measure, if properly enforced, exclude; if we can not have it without surrendering the fundamental principles upon which are based the very idea and object of the law (to suppress quackery), without the aid and co-operation of the largest element of quacks known to the profession, let us, in the name of consistency, reason and common decency, cease all efforts in that direction. For what kind of a law to "suppress quackery" would one be which places upon an equal footing with the cultivated physician a lot of *more* than quacks?—men totally uneducated in the principles of rational medicine, and who practice upon a theory long since exploded, which is contrary to reason, common sense and every-day experience? Not only have they not studied *medicine*, and are, therefore, totally ignorant of its principles and of rational therapeutics, but they have studied an absurd doctrine or theory, which inculcates the very opposite of such principles. Yet, the majority of them, as stated in a former article, while calling themselves "homeopaths," will prescribe the medicines, and employ the methods in use in the practice of rational medicine. In this, then, they are more than quacks; they are, knowingly, frauds. If there are any calling themselves homeopaths, and practicing exclusively as such; who do not resort to what they call "allopathic" medicines and treatment, thus abandoning the very principle upon which they claim their theory is founded, and attempt its very opposite (of which they are confessedly ignorant and untaught), let them consider themselves as excepted from this category; but so far as our knowledge extends, they all give up homeopathy when they encounter a serious case,—at least one, the tendency of which is to fatal termination, and, therefore, requires skill and knowledge, and resort to "strong medicine," as they call it.

The other kind of quack,—the man who has not studied medicine at all; or, take the one who has studied *at* it, and having obtained a smattering of knowledge, essays to practice; he is, at least, not a willful deception. He may be conceited enough to think he knows something about medicine, and may honestly do his best; in other words, he does not know he is an ignoramus: the other fellow does. To seriously propose, therefore, to form an alliance with the horde of homeopathic pretenders, is to sacrifice every principle to a mistaken expediency. It is the strangest argument—if so it may be called—that I ever heard emanating from a body of educated men, who notwithstanding, reiterate and affirm their allegiance to the code by a resolution which is puerile and contradictory. In their zeal to protect the people from quackery, everything else is lost sight of, and they will carry the point at any odds and by any means and at any sacrifice, even to getting the quacks to help them do it! Why not absorb all other opposition, and thus have a clear field? Let the gap down to admit one kind of cattle, and you will not be able to bar out any of the others.

It is because the writer is a friend of the Association, and in all earnestness desires to see it prosper; because he reverences its traditions and great truths,—respects the memory of its departed great—the fathers in medicine—the framers of the code—that one Palladium of the profession's honor,—that he antagonizes this measure. We earnestly protest against the step it has taken; we believe it is a mistake; one which, in later years, will be painfully recalled. It is a departure from its time-honored custom—a revolution in sentiment; and while it may be urged, as has been done, in extenuation of this step, that ideas are progressive, and that sentiment has changed (since the meeting of 1877, for instance, when the Association went on record as taking an ethical stand against affiliating with homeopaths or medical frauds), we should remember that the code has not changed, and the spirit, if not the letter of the code, clearly forbids it.

Let us abandon all idea of "regulating the practice," and ask for a law to fix a standard of requirements for medical practitioners, or to restrict the privilege of practicing medicine to those who can show to the proper authorities—and those authorities should be physicians, and there should be *one standard for all*—that they are qualified to exercise the grave prerogative, it matters not when, where nor how that qualification was obtained; make a knowledge of medicine, and a good moral character the requisite to the privilege. Let us simply ask for a bill to make quackery a penal offense; and, if we can not get it without the assistance of the homeopaths, why, give it up; but, for decency's sake, do not compromise the profession by extending to these people the long sought for recognition as physicians. (A physician is described as one skilled in the use of medicines,—and the most ardent admirer of the sect could hardly contend that prescribing infinitesimal doses of inert substances, with a view of meeting symptoms by producing similar symptoms, is skill.) Else, if we do, let us extend the same recognition to the Christian Scientists (?) and all other like absurdities, and no longer make a pretense of observing the Code of Medical Ethics.

